# ECDC’s latest publications

**DOI:** 10.2807/1560-7917.ES.2016.21.48.30412

**Published:** 2016-12-01

**Authors:** Eurosurveillance editorial team

**Affiliations:** 1 European Centre for Disease Prevention and Control (ECDC), Stockholm, Sweden

**Rapid risk and outbreak assessments**
Date of publication: 21 November 2016
Invasive cardiovascular infection by *Mycobacterium chimaera* associated with the 3T heater-cooler system used during open-heart surgery
http://ecdc.europa.eu/en/publications/Publications/RRA-mycobacterium-chimaera-November-2016.pdf
Date of publication: 18 November 2016
Outbreaks of highly pathogenic avian influenza A(H5N8) in Europe
http://ecdc.europa.eu/en/publications/Publications/risk-assessment-avian-influenza-H5N8-europe.pdf

**Scientific advice**
Date of publication: 10 November 2016
Systematic review on hepatitis B and C prevalence in the EU/EEA
http://ecdc.europa.eu/en/publications/Publications/systematic-review-hepatitis-B-C-prevalence.pdf

**Technical reports**
Date of publication: 21 November 2016
HIV testing in Europe
http://ecdc.europa.eu/en/publications/Publications/HIV-testing-guidance-evaluation.pdf

**Policy briefing**
Date of publication: 18 November 2016
Last-line antibiotics are failing: options to address this urgent threat to patients and healthcare systems
http://ecdc.europa.eu/en/publications/Publications/antibiotic-resistance-policy-briefing.pdf

**Surveillance reports**
Date of publication: 29 November 2016
HIV/AIDS surveillance in Europe 2015
http://ecdc.europa.eu/en/publications/Publications/HIV-AIDS-surveillance-Europe-2015.pdf
Date of publication: 4 November 2016
Influenza virus characterisation, Summary Europe, September 2016
http://ecdc.europa.eu/en/publications/Publications/influenza-virus-characterisation-september-2016.pdf

**Communicable Disease Threats Report**
Date of publication: every Friday
http://ecdc.europa.eu/cdtr

**Scientific peer-reviewed publications**
Updated: September 2016
http://ecdc.europa.eu/en/publications/peer-reviewed/Pages/index.aspx

